# Supplementary material for: Single Mutation in Hammerhead Ribozyme Favors Cleavage Activity with Manganese over Magnesium
Source: Noncoding RNA. 2020 Mar 20;6(1):14. doi: 10.3390/ncrna6010014 (PMC7151607; doi:10.3390/ncrna6010014)
Supplement: Supplementary file 1 [file ncrna-06-00014-s001.pdf]

Supplementary material for:

Single mutation in hammerhead ribozyme favors cleavage activity with manganese over magnesium

Mohammad Reza Naghdi[1], Emilie Boutet[1], Clarisse Mucha[1], Jonathan Ouellet[2], Jonathan Perreault[1,*]

[1]Institut National de la Recherche Scientifique (INRS), Centre Armand Frappier Santé Biotechnologie, 531 boul. des Prairies, Laval, Québec, H7V 1B7, Canada.

[2]Department of Chemistry and Physics, Monmouth University, 400 Cedar Avenue, West Long Branch, NJ, 07764, USA

* Correspondence: jonathan.perreault@inrs.iaf.ca; Tel.: (1-450-687-5010, ext. 4411);

Key words: Manganese, magnesium, cation, RNA variants, hammerhead variants

The main text presents results regarding C6 variant (Bcep176). Other ribozymes were assayed as well, some of which had cleavage activity, but with the exception of Bcep176, none of the other ribozymes were characterized further in this study. We nevertheless provide the information herein in the eventuality that it might be of interest to others.

Table S1. Ribozyme assays with different cations

	gene	Cleave during trx	Full length	Mg^{2+}	Mn^{2+}	Co^{2+}	Zn^{2+}	Cd^{2+}	Ca^{2+}	Ba^{2+}	Cu^{2+}	Ni^{2+}	Fe^{2+}	K^{+}	NH_4^{+}	Na^{+}
Bcep 176	-	-	+	-	+	-	-	-	-			-	-	-		
100	Mo	+	-/+	-	-	-	-	-								
102	Zn	-	+	-	-	-	-	-	-	-	-					
104	Zn	-	+	-	-				-	-	-	-	-			
106	NH_4	-	+	-	-	-	-	-	-	-	-	-	-	-	-	-
112	Ca	-	+	-	-	-	-	-								
115	Fe	-	+	-	-	-	-	-	-	-	-	-	-	-	-	-
117	Fe	+	-/+	-	-	-	-	-	-	-	-	-	-			
123	Cu	-	+	-	-	-	-	-	-	-	-	-				
125	Zn	-	+	-	-	-	-	-	-	-	-	-				
130	-	+	-/-	-	-	-	-	-	-	-	-	-		-	-	-
133	Cu	+	-/+	+/-	+/-	-	-	-	-	-	-	-	-	-	-	-
135	M	-	+	-	-	-	-	-	-	-	-	-	-	-	-	-
137	Cu	-	+	-	-				-			-				

Except for the Bcep176 HHRz described in the main text, the ribozyme number is the first primer number used to prepare the template of the corresponding putative ribozyme as shown in Table S2. Column "gene" corresponds to the type of metal ion to which the closest adjacent gene had an association (e.g. ribozyme 102 is found in *Branchiostoma floridae* in an intron of a Zn-finger protein), "M" refers to undefined "metal", "-" refers to no particular metal ion. "Cleave during trx": the "+" or "-" indicate whether the ribozymes cleaved or not during transcription. In the "full length" column, a "-" indicates that the full length RNA could not be isolated (because of efficient self-cleavage), a "+" means that a full length molecule could readily be isolated and a red "+" means that the full length RNA could be isolated following cleavage inhibition by addition of high concentration of a complementary oligonucleotide (or could not be isolated in the case of "-"). The following columns indicate results ("+", cleavage; "-" no cleavage) in presence of the corresponding ions (typically at 1 mM), empty space either means the ion was not assayed or that results were inconclusive (e.g. because of RNA degradation).

Table S2. Oligonucleotides used to prepare the ribozyme templates

	Oligo name	Genetic context	Oligonucleotide sequence
100	JP100_ABEF-Mo-F	Overlap cds "molybdopetrin metabolism prot" (env. seq)	TAATACGACTCACTATAggcggctgggggggcccaaacagcacgactgacgaggtc
	JP101_ABEF-Mo-R		GTATATTTGCATGAGACTGCTCGTGGCAGTTTCGGTCATTCAGACCTCGTCAGTCGTGC
102	JP102_Branchi-Zn-F	Intron Zn-finger prot. maybe Ca-dependent (*Branchiostoma floridae)*	TAATACGACTCACTATAggcaccgagtaacgttagtatatttcattttgcactgatg
	JP103_Branchi-Zn-R		TCTTCTGCAGACGTTTCGGTGACTGTCTGTCACCTTCATCAGTGCAAAATGAAATATAC
104	JP104_Branchi2-Zn-F	Idem 102	TAATACGACTCACTATAggagtatatttcattttgcactgatgaaggtgacagacagtc
	JP105_Branchi2-Zn-R		AACCTCTTTTTATTCTTCTGCAGACGTTTCGGTGACTGTCTGTCACCTTCATC
106	JP106_meta-mar-NH4-F	Overlap opposite strand NH_4^+ transporter (env. Seq)	TAATACGACTCACTATAggaaaaaaccccggtcgcctcgaccggggttcaagatccctg
	JP107_meta-mar-NH4-R		CTTACCCCGACTTCGCCTCTGCCGGTAACTGAAAGGCTCCTCAGGGATCTTGAACCC
112	JP112_Branchi5-Ca-F	Downstream of EGF-like, membrane prot. requiring Ca^{2+} (*Branchiostoma floridae)*	TAATACGACTCACTATAgggggtgggggtggtaacggcacgttggtgactgatacgtgctaagtgaccgcgaacattgaattgttct
	JP113_Branchi5-Ca-R		AAACAGAGACTTACTTCCGGACGTTTCGAGTGACATCCATCACTCTTCTTCAGGGTCACTAGAATGGACTGGCAGAACAATTCAATGTTCGCG
	JP114_Branchi5-Ca-R2		AAACAGAGACTTACTTCCGGACG
115	JP115_Desulfo-Fe-F	Downstream of Fe-dependent repressor (*Desulfovibrio vulgaris* prophage context)	TAATACGACTCACTATAggcgtgtgacgacctgacttgcgaaaccgcccccatgcggggc
	JP116_Desulfo-Fe-R		TTCCTTCGGCTGCTCGTCAGGCCGGAGCCGCCACGCTCCGACGACCGCCCCGCATGGGGG
117	JP117_Desulfo2-Fe-F	Idem 115	TAATACGACTCACTATAggcacgaagtagaagcgtagcgaaaccgcccccccatgcggg
	JP118_Desulfo2-Fe-R		TGTTCGGCTGCTCGTCAGGCCCGGCGCGCCACCGCCGGACGACCGCCCCGCATGGGGGGG
123	JP123_Bce-Cu-F	Close to copper export protein (*Bacillus cereus*)	TAATACGACTCACTATAggccctatttttcagtgtcgaaacagtttaagctgttcaatag
	JP124_Bce-Cu-R		ATTAAATGACCTATTTCTTCAGCAATAGGGAGGTCATCTCCTATTGAACAGCTTAAACTG
125	JP125_Tribo-Zn-F	Close to ATPase that requires Zn, similar to pyridoxal kinase (*Tribolium castaneum*)	TAATACGACTCACTATAggaaccggtctgcggttgtcccatgagtgtcctggcttctacaaatattccttttcgaaaactattagaggc
	JP126_Tribo-Zn-R		GACCGGTTTCGCCCTTGTTGGGGCTCATCAGTGCAGCGCAGTCCTGCGAATGTCATGGCCTCTAATA
	JP127_Tribo-Zn-R2		GACCGGTTTCGCCCTTGTTGGGGC
130	JP130_Branchi6-F	Overlap cds "molybdopetrin metabolism prot*"*, *FolK|FolB* (*Branchiostoma floridae*)	TAATACGACTCACTATAGgaagttctttaggtacaaccagggagtacttacttccaacgtttcggtagcagcgagaatcgtatccagccattctac
	JP131_Branchi6-R		AGGGTTCATTAAGCACAACCAGGAGTACTTACTTCCAACGTTTCGGTGTCTGTCAGACACCATCATCAGGGTAGAATGGCTGGATACGATTCTCG
	JP132_Branchi6-R2		AGGGTTCATTAAGCACAACCAGG
133	JP133_Ldr2-Cu-F		TAATACGACTCACTATAggaatcaagcataactgatgagcctgggtagttcaggcgaaac

	JP134_Ldr2-Cu-R	*Adjacent Cu oxidase (Legionella drancourtii probable prophage)*	GTTAGGGAACAGGATGGCTTAGCATAAGACCTTAATAAGGTTTCGCCTGAACTACCC
135	JP135_Aba-metal-F	*Metal-dependent prot (Acinetobacter baumannii ssprobably in prophage)*	TAATACGACTCACTATAggaagcataagcgaaacacaggcattcgtgcctgtgtctact
	JP136_Aba-metal-R		TCTCCCTATCGCTGCTCATCAGTACTGGATCACGACATCCAGTAGACACAGGCACGAATG
137	JP137_Spr-Cu-F	*Adjacent Cu homeostasis (Serratia proteamaculans)*	TAATACGACTCACTATAggccgatgagcgtctggtgtttaccaaaggcctgagcgccg
	JP138_Spr-Cu-R		AGCCCGTTATGATCGTTTCGCCGCCAGATTTCCGGCTCATCATCGGCGCTCAGGCCTTTG

Bases in uppercase of forward primers correspond to T7 promoter. Sequences are taken from (Perreault et al. 2011).

A

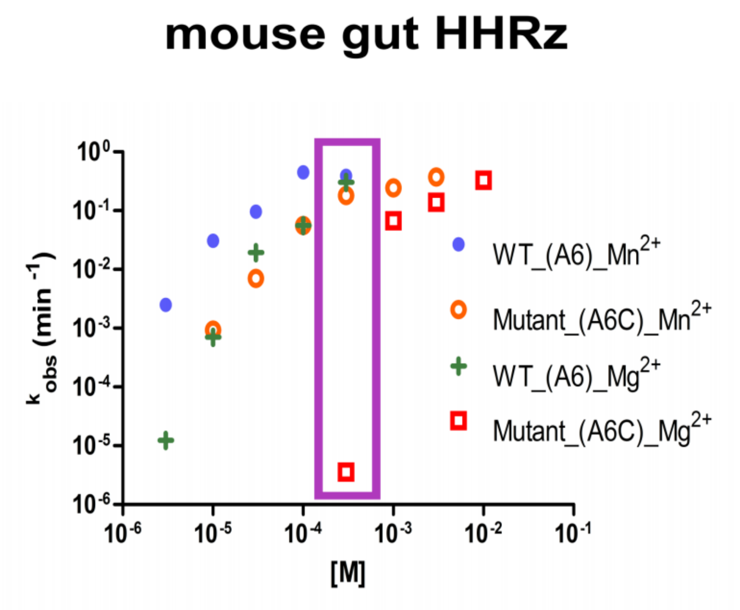

B

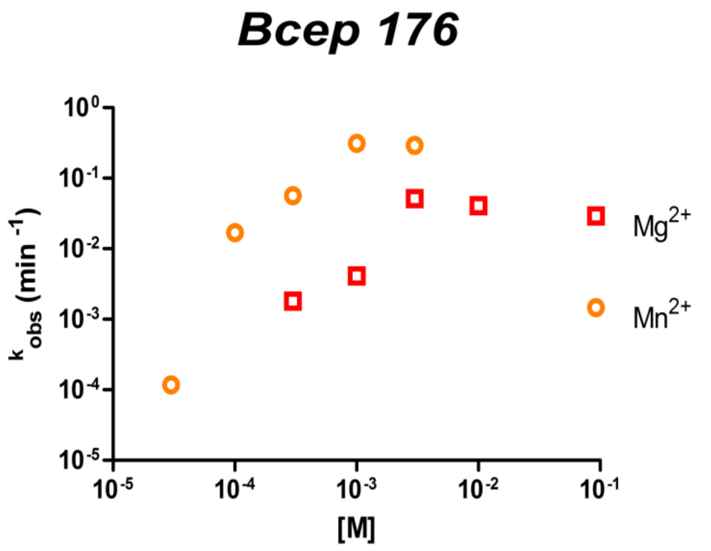

Figure S1: Comparison of cleavage activity of type II hammerhead ribozyme from gut of mouse, mouse gut HHRz : wild type (A6) versus mutant (C6) and *Bcep176* (C6). (A) The obtained k_{obs} from mouse gut HHRz were plotted against the concentration. The violet box indicates how mutated version in presence of Mg^{2+} drastically loses cleavage activity at 300 µM, whereas a similar cleavage rate was observed for WT. (B) The obtained k_{obs} for *Bcep176* (C6) were plotted against the concentration. The graph presents better k_{obs} for Mn^{2+}over Mg^{2+}. Yet, we could not determine an improvement in affinity for Mn^{2+} of the C6 versions compared to A6, but note that attempts to determine affinities for Mg^{2+} and Mn^{2+} did not provide conclusive results (the calculated K_{Mg} and K_{Mn} had standard errors much larger than calculated constants).
